# Excess risk of chronic physical conditions associated with depression and anxiety

**DOI:** 10.1186/1471-244X-14-10

**Published:** 2014-01-16

**Authors:** Rituparna Bhattacharya, Chan Shen, Usha Sambamoorthi

**Affiliations:** 1 Department of Pharmaceutical Sciences and Policy, School of Pharmacy, West Virginia University, P.O. Box 9510, Morgantown, WV 26506-9510, USA; 2 Center For Cardiovascular And Respiratory Sciences, West Virginia University, Morgantown, WV, USA; 3 Department of Biostatistics, University of Texas, MD Anderson Cancer Center, Houston, Texas, USA

**Keywords:** Depression, Anxiety, Obesity, Smoking, Physical activity, Chronic physical conditions

## Abstract

**Background:**

Depression and anxiety have been reported to be associated with chronic physical conditions. We examined the excess risk of chronic physical conditions associated with depression and/or anxiety within a multivariate framework controlling for demographic and modifiable lifestyle risk factors.

**Methods:**

We used a retrospective cross-sectional study design. Study participants were adults aged 22–64 years from 2007 and 2009 Medical Expenditure Panel Survey. We defined presence of depression-anxiety based on self-reported depression and anxiety and classified adults into 4 groups: 1) depression only; 2) anxiety only; 3) comorbid depression and anxiety 4) no depression and no anxiety. We included presence/absence of arthritis, asthma, chronic obstructive pulmonary disorder, diabetes, heart disease, hypertension, and osteoporosis as dependent variables. Complementary log-log regressions were used to examine the excess risk associated with depression and/or anxiety for chronic physical conditions using a multivariate framework that controlled for demographic (gender, age, race/ethnicity) and modifiable lifestyle (obesity, lack of physical activity, smoking) risk factors. Bonferroni correction for multiple comparisons was applied and p ≤0.007 was considered statistically significant.

**Results:**

Overall, 7% had only depression, 5.2% had only anxiety and 2.5% had comorbid depression and anxiety. Results from multivariable regressions indicated that compared to individuals with no depression and no anxiety, individuals with comorbid depression and anxiety, with depression only and with anxiety only, all had higher risk of all the chronic physical conditions. ARRs for comorbid depression and anxiety ranged from 2.47 (95% CI: 1.47, 4.15; P = 0.0007) for osteoporosis to 1.64 (95% CI: 1.33, 2.04; P < 0.0001) for diabetes. Presence of depression only was also found to be significantly associated with all chronic conditions except for osteoporosis. Individuals with anxiety only were found to have a higher risk for arthritis, COPD, heart disease and hypertension.

**Conclusion:**

Presence of depression and/or anxiety conferred an independent risk for having chronic physical conditions after adjusting for demographic and modifiable lifestyle risk factors.

## Background

Depression and anxiety have been reported to be associated with chronic physical conditions [1]. Evidences from cross-sectional, longitudinal studies, systematic reviews, and meta-analyses have concluded that depression is an independent risk factor for developing chronic physical conditions [2-9]. Published research has shown that depression can lead to type 2 diabetes (hereafter referred to as diabetes) [3], cardiovascular diseases (CVD) [4] and hypertension [5]. In a Canadian study it was found that among young adults between 20 and 50 years, prior history of depression was related to new onset diabetes [10]. Results from meta-analyses showed that individuals with depression had a 64% increased risk of development of heart disease [11] and 60% increased risk of diabetes [12]. Several studies also have reported depression as a significant predictor of osteoporosis and fractures [6-9]. An association between depression and Chronic Obstructive Pulmonary Disease (COPD) is established through an indirect pathway via increased nicotine dependence among those with depression. [13-15].

However, there has been mixed findings on the association between anxiety and risk for some chronic physical conditions. A prospective association between anxiety and risk of hypertension has been reported [16]. A Danish study also revealed an increased likelihood of hypertension among those with anxiety disorders [17]. The association between anxiety and risk of developing diabetes has been inconclusive. While some studies showed an increased risk of diabetes among those with anxiety as compared to those without anxiety [18], other studies have not shown any association between anxiety and diabetes [19]. Studies have reported an indirect association between anxiety and osteoporosis; compared to adults without anxiety, those with anxiety had lower hip bone mass density [20], which is a risk factor for osteoporosis [21]. A recently concluded meta-analysis indicated that depression or anxiety results in a 43% increased incidence or exacerbation risk of COPD, particularly among non-elderly adults [22].

In addition, depression and anxiety co-occur in more than 50% of individuals with depression [23-25]. To the best of our knowledge, only one study using mental health survey in 17 countries addressed the association between comorbid depression and anxiety on risk for chronic physical conditions [26]. This study concluded that comorbid depression and anxiety was associated with increased likelihood of 10 different chronic physical conditions (asthma, arthritis, back/neck problems, chronic headache, diabetes, heart disease, hypertension, multiple pains, obesity and ulcer). For example, the age and gender adjusted odds ratios (AOR) and 95% Confidence Interval (CI) for comorbid depression and anxiety was 1.6(1.4, 1.9) for asthma 2.5(2.2, 2.9) for arthritis and 2.8(2.3, 3.4) for heart disease, compared to those without depression and without anxiety.

A general limitation of many of the studies described above has been the lack of comprehensive controls when analyzing the association between depression, anxiety and chronic physical conditions. Individuals with depression and/or anxiety also have higher rates of obesity [27,28], smoking [29,30] and lower rates of physical activity [31] compared to those without depression and without anxiety. In addition, studies have documented that modifiable lifestyle risk factors such as obesity, lack of physical activity, and smoking can contribute to the increased risk for chronic physical conditions [32-34]. Therefore it is very important to control for these risk factors in order to assess the excess risk of chronic physical conditions associated with the presence of depression and anxiety.

The primary purpose of our study was to examine excess risk of a diverse set of chronic physical conditions associated with depression and/or anxiety in a non-elderly adult population within a multivariate framework that controlled for demographic and modifiable lifestyle risk factors. We focus on the non-elderly adult population because individuals with depression and/or anxiety may develop chronic physical conditions at an earlier age as compared to general population [2]. We hypothesize that even after controlling for these risk factors individuals with depression and/or anxiety will have a greater risk of arthritis, asthma, COPD, diabetes, heart disease, hypertension and osteoporosis as compared to individuals with neither depression nor anxiety.

## Methods

### Study design

We used a retrospective cross-sectional study design using data from a nationally representative household survey in the United States (US).

### Data source and setting

The current study utilized data from the annual releases of 2007 and 2009 Medical Expenditure Panel Survey (MEPS), a nationally representative annual survey of households representing the US non-institutionalized civilian population. The survey adopted a multistage, clustered sample design [35].

The household component (HC) is the core of the MEPS data. Extensive health care utilization data, including demographics, health conditions, health status, use of medical care and prescription medications, detailed charges and payment, insurance coverage, income, and employment are captured in the HC through annual computer-assisted in-person (CAPI) interviews. Information on medical conditions can be derived from the Medical Conditions file of the HC of MEPS. Details of how medical conditions were derived from these files are provided in the Measures section below.

### Analytic sample

We restricted our analytical sample to individuals who were between the ages of 22 and 64 years, were not underweight (i.e. BMI <18.5 kg/m^2^), and were alive as of the end of the calendar years 2007 and 2009. Based on these selection criteria, participants from 2007 and 2009 MEPS data were pooled together to yield a final sample of 33,242 participants. In our study sample, a very small number (only 1.2%) of adults were in the underweight BMI group and therefore we excluded underweight BMI group from our analytic sample. Individuals with self-reported schizophrenia, psychoses, attention deficit hyper activity disorders, adjustment disorders, substance abuse disorders and personality disorders were excluded from analysis.

### Measures

#### *Dependent variables:* presence/absence of chronic physical conditions

As mentioned above, chronic physical conditions were derived from medical conditions file. The MEPS investigators followed many procedures to minimize under-reporting of medical and mental health conditions. Conditions can be reported: (1) in the Priority Condition Enumeration section in which respondents are asked if they have been diagnosed by a doctor or other health care professional with specific conditions. The priority conditions for 2007 and 2009 included (i) long term life threatening conditions like Cancer, Hypertension/High Blood Pressure, Diabetes/Sugar Diabetes, High Cholesterol Ischemic Heart Disease, Stroke, HIV/AIDS and Emphysema (ii) chronic manageable conditions like Arthritis, Chronic Bronchitis, Asthma, Joint Pain, Gall Bladder Diseases, Stomach Ulcers, Back Problems of any kind and (iii) mental health issues like Alzheimer’s Disease and Other Dementias, depression and anxiety, Attention Deficit Hyperactivity Disorder (ADHD)/Attention Deficit Disorder (ADD) (2) if the respondents sought care for a reported condition (hospital stay, outpatient visit, emergency room visit, home health episode, prescribed medication purchase, or medical provider visit) (3) if the respondents reported one or more episodes of disability days due to a specific condition and (4) if the condition was “bothering” the person during the reference period. Health problems as defined by MEPS refers to “physical conditions, accidents, or injuries that affect any part of the body as well as mental or emotional health conditions, such as feeling sad, blue, or anxious about something.” These self-reported conditions are then mapped to International Classification of Diseases (ICD-9-CM) diagnosis codes and further recoded to aggregate clinical classification codes (CCCODEX) by MEPS researchers. The cross-walk file between ICD-9-CM codes and clinical classification codes are published [36]. In order to minimize bias associated with self-reported data, upon completion of the household CAPI interview and obtaining permission from the household survey respondents, a sample of medical providers are contacted by telephone to obtain information that household respondents could not accurately provide [35,37].

In this paper, chronic physical conditions consisted of self-reported diagnosis of asthma, COPD, diabetes, heart disease, hypertension, arthritis and osteoporosis. Heart disease included heart valve disorders, peri- endo-, and myocarditis, cardiomyopathy, acute myocardial infarction, coronary atherosclerosis and other heart disease, non-specific chest pain, pulmonary heart disease, conduction disorders, cardiac dysrhythmias, congestive heart failure and other and ill-defined heart disease. Arthritis included Infective arthritis and osteomyelitis (except that caused by tuberculosis or sexually transmitted disease), rheumatoid arthritis and related disease, osteoarthritis and other non-traumatic joint disorders. These chronic physical conditions were selected based on prevalence, economic, morbidity and mortality burden [38]. We used three-digit International Classification of Diseases-9th edition-Clinical Modification (ICD-9-CM) and clinical classification codes provided in the medical care event files to identify presence of these conditions.

#### Key independent variable: depression-anxiety status

Presence of depression and/or anxiety was derived from the Medical Conditions file. We used clinical classification codes to identify depression (code: 657) and anxiety (code: 651). Based on the presence of depression and anxiety we categorized the individuals into 4 groups: 1) Depression only (individuals with only depression and no anxiety); 2) Anxiety Only (individuals with only anxiety and no depression); 3) Comorbid depression and anxiety (individuals with both depression and anxiety); and 4) No depression and no anxiety (individuals with neither depression nor anxiety).

#### Other independent variables

We also included variables that may be associated with risk for chronic physical conditions. These included demographic factors (gender, age, race/ethnicity, and metro status), socio-economic status (education and poverty status), access to care (health insurance), and modifiable life-style risk factors. Modifiable life-style risk factors were overweight and obesity as measured by Body Mass Index (BMI) values, lack of physical activity, current smoking status. BMI was grouped into three categories: normal BMI (18.5 kg/m2-24.9 kg/m2); overweight (25 kg/m2-29.9 kg/m2); and obese (≥ 30 kg/m2 ) [39]. Lack of physical activity was measured as a binary variable based on vigorous activity at least three days a week (Yes/No). Current smoking status was grouped as current smoker, former smoker, and never smoked.

### Statistical techniques

Significant bivariate group differences in the association between depression-anxiety status and chronic physical conditions were tested with chi-square statistic. Logistic regressions are commonly used to estimate the association between binary dependent variable and other independent variables. However, the AORs from logistic regressions cannot be interpreted as risk when the disease prevalence is high (i.e. >10%), as is the case for many of our chronic conditions, since AORs fail to give approximations for risk [40]. Simulation studies have shown that complementary log-log (CLL) regression models can be used as an alternative statistical model for estimating multivariable-adjusted prevalence ratios and their confidence intervals [41]. The CLL regression models can be easily implemented with standard statistical software packages such as Statistical Analysis Software (SAS). In the current study, coefficients from CLL regressions were exponentiated to summarize the excess risk of chronic physical conditions associated with depression-anxiety status [42]. The CLL regressions included all the independent variables mentioned in the “Measures” section. For our key independent variable, depression-anxiety status, “no depression and no anxiety” group was used as the reference category. All analyses accounted for the complex design of MEPS. As we tested our hypothesis for seven different chronic physical conditions, the significance level for multiple comparisons was controlled by applying the Bonferroni correction for multiple comparisons and p ≤ 0.007 was considered statistically significant. Analyses were performed in SAS 9.3 [43].

## Results

In our study sample of young adults between the ages of 22 and 64 years (data not presented in tabular form), majority of the study sample were white (66%), women (51%), lived in a metro area (85%), and had private insurance (73%). Forty-eight percent had college education and 43% had incomes representing 400% or more federal poverty line. Nearly 1 in five individuals were current smokers and 60% exercised more than 3 time each week; overall, 31% reported a normal BMI; 35% were classified as overweight; and 30% were categorized as obese based on BMI values.

Table 1 presents the distribution of study sample characteristics by depression-anxiety anxiety status. Overall, 7% had depression only, 5% had anxiety only and 3% had comorbid depression and anxiety. We observed significant group differences in depression-anxiety status by all the variables included in the study. A higher proportion of women (3.7%) than men (1.6%) reported comorbid depression and anxiety compared to no depression and no anxiety. A higher proportion of individuals with comorbid depression and anxiety were poor compared to those who had high income (4.8% vs. 1.9%), were obese as compared to those with normal weight (3.6% vs. 2.4%), were current smokers compared to individuals who never smoked or were former smokers (4.5% vs. 2.3%). Similar findings were noted for depression only and anxiety only categories.

**Table 1:** Description of study sample by depression-anxiety status medical expenditure panel survey, 2007-2009

	**Depression only**		**Anxiety only**		**Comorbid depression and anxiety**		**No depression and no anxiety**		
	**N**	**Wt%**	**N**	**Wt%**	**N**	**Wt%**	**N**	**Wt%**	**P-value**
**ALL**	2,326	7.3	1,719	5.6	849	2.7	28,348	84.4	
**Gender**									< .0001
Women	1,610	9.5	1,121	7.1	618	3.7	14,452	79.7	
Men	716	5.2	598	4.0	231	1.6	13,896	89.2	
**Race/ethnicity**									< .0001
White	1,454	8.6	1,065	6.5	568	3.2	12,704	81.8	
African American	309	4.7	214	3.5	95	1.7	5,256	90.1	
Latino	454	5.3	332	4.1	145	1.6	7,845	89.1	
Others	109	4.6	108	3.9	41	1.6	2,543	89.9	
**Age in years**									< .0001
21-39	749	5.6	663	5.2	265	2.0	12,414	87.2	
40-49	633	7.9	449	5.7	217	2.8	7,199	83.5	
50 – 64	944	9.1	607	6.0	367	3.4	8,735	81.5	
**Metro status**									0.0048
Metro	1,919	7.1	1,466	5.6	698	2.6	24,468	84.7	
Not Metro	407	8.8	253	5.4	151	3.1	3,880	82.8	
**Education**									0.0063
Less than high school	505	8.1	306	5.0	196	3.1	5,725	83.8	
High school	748	8.0	498	5.5	253	2.8	8,629	83.7	
College	1,063	6.8	911	5.8	395	2.5	13,724	84.8	
**Health insurance**									< .0001
Private	1,347	6.8	1,204	5.9	460	2.3	18,452	85	
Public	566	15	255	6.7	287	8.2	2,973	70	
Uninsured	413	6.2	260	3.7	102	1.7	6,923	88.4	
**Poverty status**									< .0001
Poor	515	10.5	271	5.9	234	4.8	4,222	78.7	
Near poor	525	8.4	305	5.1	173	3.2	5,811	83.4	
Middle income	625	6.7	561	5.9	243	2.7	8,897	84.6	
High income	661	6.6	582	5.4	199	1.9	9,418	86.1	
**Body mass index categories**									< .0001
Normal	603	6.0	517	5.7	238	2.4	8,830	85.9	
Overweight	688	6.3	559	5.0	243	2.1	10,120	86.5	
Obese	980	10.0	602	6.1	351	3.6	8,520	80.2	
**Smoking status**									< .0001
Current smoker	650	10.2	415	7.0	284	4.5	5,011	78.3	
Former/never smoked	1,529	6.7	1,216	5.4	529	2.3	21,037	85.6	
**Physical activity**									< .0001
Vigorous activity at least 3 days/week	1,062	5.8	916	5.2	360	2.0	16,394	86.9	
Other	1,255	9.7	796	6.3	486	3.7	11,630	80.3	

*Note:* Study sample comprised of adults aged 22–64 years alive during the calendar year without self-reported diagnosis of schizophrenia, psychoses, attention deficit hyper activity disorders, adjustment disorders, substance abuse disorders and personality disorders.

Wt: Weighted.

Significant if p < =0.007.

Rates of chronic physical conditions by depression-anxiety status are presented in Table 2. The percentage of individuals with chronic physical conditions was highest in the group with comorbid depression and anxiety, followed by those with only depression only, anxiety only and those with no depression and no anxiety. Individuals with comorbid depression and anxiety had the highest prevalence of arthritis (39%) followed by hypertension (35%), COPD (21%), heart disease (17%), diabetes (15%), asthma (14%) and osteoporosis (4%).

**Table 2:** Number and weighted percentage of chronic physical conditions by depression-anxiety status medical expenditure panel survey, 2007-2009

	**Depression only**		**Anxiety only**		**Comorbid depression and anxiety**		**No depression and no anxiety**		
	**N**	**Wt%**	**N**	**Wt%**	**N**	**Wt%**	**N**	**Wt%**	**P-value**
**Arthritis**	831	34.3	481	27.2	360	38.8	4,114	14.8	< .0001
**Asthma**	258	10.2	131	7.2	131	13.8	1,195	4.2	< .0001
**COPD**	403	16.7	235	13.7	196	21.3	1,925	7.1	< .0001
**Diabetes**	348	13.2	165	8.2	139	14.7	2,063	6.2	< .0001
**Heart disease**	301	11.2	195	10.5	149	16.9	1,449	5.3	< .0001
**Hypertension**	786	30.6	470	25.5	313	34.5	5,092	16.9	< .0001
**Osteoporosis**	50	2.1	32	2.0	26	3.5	199	0.8	< .0001

Note: Study sample comprised of adults, 22–64 years of age, alive during the calendar year and without self-reported diagnosis of schizophrenia, psychoses, attention deficit hyper activity disorders, adjustment disorders, substance abuse disorders and personality disorders.

COPD: Chronic Obstructive Pulmonary Disease; Wt: Weighted.

Significant if p < =0.007.

In Table 3, we present the adjusted risk ratios (ARRs) and 95% Confidence intervals (CI) from CLL regressions for depression-anxiety status after controlling for demographic, socio-economic, access to care and modifiable life-style risk factors. Results from CLL regressions indicated that compared to individuals with no depression and no anxiety, groups with comorbid depression and anxiety had greater risk of all chronic physical conditions. The ARRs for comorbid depression and anxiety ranged from 2.47 (95% CI: 1.47, 4.15; P = 0.0007) for osteoporosis to 1.64 (95% CI: 1.33, 2.04; P < 0.0001) for diabetes. Presence of depression only was also found to be significantly associated with all chronic conditions except for osteoporosis. ARRs ranged 1.94 (95% CI: 1.75, 2.14; P < 0.0001) for arthritis to 1.49 (95% CI: 1.36, 1.64; P < 0.0001) for heart disease. Individuals with anxiety only were found to have a higher risk for arthritis (ARR: 1.71, 95% CI: 1.54, 1.90; P < 0.0001), COPD (ARR: 1.62, 95% CI: 1.37, 1.91; P < 0.0001), heart disease (ARR: 1.74, 95% CI: 1.45, 2.09; P < 0.0001) and hypertension (ARR: 1.48, 95% CI: 1.31, 1.68; P < 0.0001).

**Table 3:** **Associations between depression-anxiety status and chronic physical conditions**^**a**^**adjusted risk ratios (ARR) and 95% confidence intervals medical expenditure panel survey, 2007 and 2009**

	**Depression only**			**Anxiety only**			**Comorbid**		
							**Depression and anxiety**		
	**ARR**	**[95% CI]**	**P-value**	**ARR**	**[95% CI]**	**P-value**	**ARR**	**[95% CI]**	**P-value**
**Arthritis**	1.94	[1.75,2.14]	<0.0001	1.71	[1.54,1.90]	<0.0001	2.04	[1.79,2.33]	<0.0001
**Asthma**	1.78	[1.50,2.11]	<0.0001	1.4	[1.13,1.75]	0.0023	2.16	[1.66,2.81]	<0.0001
**COPD**	1.76	[1.54,2.02]	<0.0001	1.62	[1.37,1.91]	<0.0001	2.05	[1.68,2.49]	<0.0001
**Diabetes**	1.59	[1.37,1.84]	<0.0001	1.21	[1.00,1.47]	0.0457	1.64	[1.33,2.02]	<0.0001
**Heart disease**	1.56	[1.34,1.81]	<0.0001	1.74	[1.45,2.09]	<0.0001	2.16	[1.77,2.63]	<0.0001
**Hypertension**	1.49	[1.36,1.64]	<0.0001	1.48	[1.31,1.68]	<0.0001	1.66	[1.44,1.93]	<0.0001
**Osteoporosis**	1.78	[1.22,2.61]	0.0433	1.6	[1.01,2.53]	0.0029	2.47	[1.47,4.15]	0.0007

*Note:* Analytic sample comprised of adults, 22–64 years of age with no self-reported diagnosis of schizophrenia, psychoses, attention deficit hyper activity disorders, adjustment disorders, substance abuse disorders and personality disorders.

Reference group for multivariable complementary log-log regression estimates: “no depression and no anxiety

^a^Model Controlled for modifiable lifestyle risk factors (weight-status, physical activity, smoking status), age, gender, race/ethnicity, education, whether residing in a metro area (yes/no) and health insurance.

COPD = Chronic Obstructive Pulmonary Disease.

Significant if P < =0.007.

### Secondary analyses

It is plausible that use of psychotropic medications may be independently associated with higher risk of chronic physical conditions compared to those without psychotropic medications use. For example, Paroxetine, a selective serotonin reuptake inhibitors often used to treat depression may hamper the function of vascular endothelial cells by impairing endothelial nitric oxide synthase, thus affecting arterial integrity leading to cardiovascular diseases [44]. Tricyclic antidepressants can affect cardiac conduction and rhythm, and could be cardiotoxic [45] leading to greater risk of developing heart disease. Elevated risk for glucose dysregulation, weight gain and new onset diabetes has been observed with the use of antipsychotic and antidepressants medications [46-48]. Both typical and atypical antipsychotic medications are known to induce hyperprolactinemia [49] which leads to bone loss, a major risk factor for osteoporosis [50]. We tested this by additionally including psychotropic medication use in the complementary log-log regression models. Psychotropic medication use was identified from prescription drug files using Multum Lexicon therapeutic categories (category code = 242).

Overall, 11.4% of the study sample used psychotropic medications; 80% of those with comorbid depression and anxiety, 67% of those with depression only, 40% of those with anxiety only and 2.5% of those with no depression and no anxiety had psychotropic medication use. Among those with comorbid depression and anxiety, non-users of psychotropic medication varied by type of chronic physical conditions; among those with diabetes and comorbid depression and anxiety, only 0.4% did not use psychotropic medications; similar findings were noted for hypertension (0.6%), arthritis (0.9%), COPD (1.1%), heart disease (1.2%), arthritis (1.5%) and osteoporosis (1.5%),

When we additionally included use of psychotropic medications along with other independent variables mentioned in the measures section, we found that psychotropic medication use was independently associated with increased risk of arthritis (ARR: 1.28; 95% CI: 1.15-1.42; P < 0.0.0001), diabetes (ARR: 1.51; 95% CI: 1.27-1.78; P < 0.0.0001) and hypertension (ARR: 1.23; 95% CI: 1.13-1.42; P < 0.0.0001).

The association between depression-anxiety status and higher risk of chronic physical conditions remained the same except for diabetes. The association between comorbid depression and anxiety and osteoporosis was no longer statistically significant after controlling for psychotropic medication use. However, one needs to use extreme caution in interpreting these results, because of the very high correlation between psychotropic medication use and depression anxiety status variable (> 0.66) and very small sample size in comorbid depression and anxiety and no psychotropic medication for some of the chronic conditions. Results from these regression models are available from the first author upon request.

## Discussion

Our paper examined the excess risk of chronic physical conditions associated with depression and/or anxiety among non-elderly adults after controlling for a comprehensive list of demographic, socio-economic, access to care, and modifiable lifestyle risk factors for chronic conditions. In our study sample of non-elderly adults 15.6% had either depression or anxiety or both. In multivariable regressions, we found that depression-anxiety status was associated with greater risk of asthma, arthritis, COPD, diabetes, heart disease, hypertension, and osteoporosis. While our research focused on the one-way relationship between depression-anxiety status and risk of chronic conditions, one cannot rule out bidirectional associations. Bidirectional association between depression and/or anxiety and risk of many medical illness such as diabetes [1], hypertension, cardiovascular diseases [1,51], COPD [22,52], asthma [52], arthritis [53] and osteoporosis [54] has been documented. However, owing to the cross-sectional design used in the study, we were unable to test any bidirectional associations between depression-anxiety status and the risk of chronic physical conditions.

Although not directly analyzed in this study, we can speculate on the reasons for excess risk for chronic physical conditions associated with depression and/or anxiety. Prior research has attributed this excess risk to biological factors [44], use of psychotropic medications [44] as well as access to care issues [55]. Several biological mechanisms including decreased heart rate variability [56,57], increased platelet aggregation [58], higher levels of inflammatory risk markers (C-reactive protein and interleukin-6) [59] among individuals with depression and/or anxiety may lead to increased risk for cardiovascular diseases. Hypothalamic-pituitary-adrenocortical (HPA) axis dysregulation and sympathetic nervous system stimulation associated with glucose homeostasis are suggested explanations for the observed association of diabetes with depressive symptoms and major depression [60]. The pathogenic mechanisms of COPD and depression and/or anxiety are complex. Common symptomologies such as shortness of breath or dyspnea, and hyperventilation are observed in individuals with COPD as well as panic anxiety [61]. Furthermore, both anxiety and depression are associated with activation of the HPA axis and increased systemic inflammatory responses which may be responsible for increased risk of COPD exacerbations [62-66]. Dysregulation of certain stress-sensitive biological processes such as the autonomic nervous system, that contribute to the pathophysiology of both conditions, may explain the association between depression and/or anxiety and asthma [67]. The association between arthritis and depression can be attributed to the relationship between depression and inflammation; various inflammation markers such as C-reactive proteins are positively associated with depression [68]. The association between depression and low bone mineral density certainly helps to explain the risk of osteoporosis in this population [9].

Individuals with depression and/or anxiety may face a wide array of patient-, provider- and system-level barriers, which can restrict access to needed medical care. Such restricted healthcare access may increase the risk of chronic physical conditions. It has been reported that individuals with depression had problems with all four domains of primary care namely access, comprehensiveness, co-ordination and continuity of care [10]. Again individuals with anxiety often lack planned follow up and monitoring, which can be a barrier for effective preventive healthcare to reduce the risk of developing chronic conditions [69]. As primary care use have been shown to be effective in modifying the risk factors for chronic physical conditions [70], lack of appropriate primary care among individuals with depression and/or anxiety can also lead to development of chronic physical conditions. The emerging healthcare delivery models such as patient-centered medical homes and accountable care organizations, in which emphasis has been placed on primary care, [71] may facilitate efforts to reduce the risk of chronic physical conditions. Future research needs to evaluate the success of these emerging healthcare delivery models in effectively reducing the risk of chronic physical conditions in vulnerable individuals with depression and/or anxiety.

Findings from our study indicated that individuals with depression and/or anxiety are more likely to suffer from poor physical health, high rates of obesity and smoking. These findings highlight the need for integrating wellness efforts within mental healthcare settings. In addition, emphasis on physical wellness needs to be part of broader efforts to improve mental health [72]. In order to improve both physical and mental health, primary care services need to be integrated within mental health settings (reverse integration). Randomized trials and experimental studies have provided evidence on the beneficial effects of integrated primary care and mental health care for patients with depression and/or anxiety [73]. In addition to wellness efforts, routine screening for depression and/or anxiety along with co-ordinated mental-health care for those who screen positive for depression and/or anxiety may reduce the risk of chronic physical conditions.

The study had a number of strengths, including nationally representative sample, comprehensive list of independent variables that may affect chronic physical conditions and the ability to analyze the association between depression-anxiety status and a number of chronic physical conditions. We were also able to assess the independent effects of depression only, anxiety only, and comorbid depression and anxiety on risk of chronic conditions. Limitations have to be considered when interpreting the findings of the present study. The study design is retrospective, observational, and cross-sectional and therefore cannot establish causal relationships. Study variables are based on self-reported data which might be subject to reporting-bias. As our study assessed the excess risk of chronic physical conditions among non-elderly adults, the study results are not generalizable to other age groups. Although we adjusted for a large number of risk factors that may affect presence of chronic conditions, some variables such as family history that could contribute to the excess risk of chronic physical conditions were not available. Finally, our study was not immune to the inherent limitations associated with use of secondary data such as measurement errors, errors in data editing and imputation, sampling and non-sampling errors, reporting errors and interviewer effects.

## Conclusions

Despite the limitations, our study has made a unique contribution to the literature by analyzing the independent contribution of depression, anxiety, and comorbid depression and anxiety on the risk of having chronic physical conditions, after controlling for demographic, life-style, and other important risk factors. Our study findings confirmed that even after adjusting for modifiable lifestyle risk factors, depression and/or anxiety made an independent contribution to the excess risk for many chronic physical conditions. Our study findings highlight the need to minimize the burden of depression and anxiety either through better surveillance, monitoring and treatment of these conditions, which may in turn contribute to reduction in the risk of developing chronic physical conditions.

## Competing interests

Financial competing interests

1. In the past five years have you received reimbursements, fees, funding, or salary from an organization that may in any way gain or lose financially from the publication of this manuscript, either now or in the future? Is such an organization financing this manuscript (including the article-processing charge)? If so, please specify.

No

2. Do you hold any stocks or shares in an organization that may in any way gain or lose financially from the publication of this manuscript, either now or in the future? If so, please specify.

No

3. Do you hold or are you currently applying for any patents relating to the content of the manuscript? Have you received reimbursements, fees, funding, or salary from an organization that holds or has applied for patents relating to the content of the manuscript? If so, please specify.

No

4. Do you have any other financial competing interests? If so, please specify.

No

Non-financial competing interests

5. Are there any non-financial competing interests (political, personal, religious, ideological, academic, intellectual, commercial or any other) to declare in relation to this manuscript? If so, please specify.

No

## Authors’ contributions

RB participated in conceptualization, study design, statistical analysis and drafting the manuscript. CS helped in refining the study design, participated in writing of the manuscript and provided feed-back on successive iterations of the manuscript draft. US conceived the study, helped refining study design, participated in statistical analysis and provided feed-back in successive manuscript drafts. All authors read and approved the final manuscript.

## Pre-publication history

The pre-publication history for this paper can be accessed here:

http://www.biomedcentral.com/1471-244X/14/10/prepub
